# Is Parenting Associated with Teenagers' Early Sexual Risk-Taking, Autonomy And Relationship with Sexual Partners?

**DOI:** 10.1363/4303011

**Published:** 2011-01-10

**Authors:** Alison Parkes, Marion Henderson, Daniel Wight, Catherine Nixon

**Affiliations:** Sexual Health and Families Team, Medical Research Council Social and Public Health Sciences UnitGlasgow, Scotland

## Abstract

**CONTEXT:** Extensive research has explored the relationship between parenting and teenagers’ sexual risk-taking. Whether parenting is associated with wider aspects of teenagers’ capacity to form satisfying sexual relationships is unknown.

**METHODS:** Self-reported data were collected in 2007 from 1,854 students, whose average age was 15.5 years, in central Scotland. Multivariate analyses examined associations between parenting processes and sexual outcomes (delayed first intercourse, condom use and several measures reflecting the context or anticipated context of first sex).

**RESULTS:** Parental supportiveness was positively associated with all outcomes (betas, 0.1–0.4), and parental values restricting intercourse were positively associated with all outcomes except condom use (0.1–0.5). Parental monitoring was associated only with delayed intercourse (0.2) and condom use (0.2); parental rules about TV content were associated with delayed intercourse (0.7) and expecting sex in a relationship, rather than casually (0.8). Frequency of parental communication about sex and parental values endorsing contraceptive use were negatively associated with teenagers’ delayed intercourse (–0.5 and –0.3, respectively), and parents’ contraceptive values were negatively associated with teenagers’ expecting sex in a relationship (–0.5). Associations were partly mediated by teenagers’ attitudes, including value placed on having sex in a relationship.

**CONCLUSIONS:** Parents may develop teenagers’ capacity for positive and safe early sex by promoting skills and values that build autonomy and encourage sex only within a relationship. Interventions should promote supportive parenting and transmission of values, avoid mixed messages about abstinence and contraception, and acknowledge that teenagers may learn more indirectly than directly from parents about sex.

Research during the last two decades on the effect of family processes on teenage sexual behavior has focused almost exclusively on whether positive parenting may delay first sex and reduce sexual risk-taking.^1^–^5^ However, little evidence exists about possible associations between parenting and the development of attitudes, skills and opportunities that are conducive to teenagers’ or young adults’ ability to establish satisfying sexual relationships.

The current direction of research on family processes in relation to teenage sexual behavior reflects both public health concerns and theoretical approaches to the development of risk behaviors, such as problem behavior^6^ and social control theories.^7^ Some scholars have called for a change in perspective, to focus on the construction of sexuality as a developmental task of adolescence and early adulthood, and to take account of the wider emotional and social components of sexual health encompassed by the World Health Organization* definition.^8^–^10^ Identity, intimacy and autonomy are viewed as essential to the formation of positive adolescent sexuality.^3^ Here, we focus on autonomy and relatedness. Self-determination theory and similar concepts suggest that these are critical aspects of adult functioning, or optimal capabilities, which positive parenting might encourage.^11^–^13^ In the context of sexual relationships, we use “sexual autonomy” to refer to volition and control in sexual decision making, and “sexual relatedness” to having sex in the context of an established, rather than casual, relationship. We investigate these outcomes not primarily because of their demonstrated associations with lower sexual risk-taking,^14^,^15^ but because they are associated with higher quality sexual experiences among teenagers.^16^–^18^ We are, therefore, concerned with indirect, rather than direct, effects of parenting on the quality of early teenage sexual behavior, although some evidence suggests more direct associations.^19^,^20^ In fostering a teenager’s development of optimal capabilities, parents are likely to promote both positive and safe early sexual relationships. Sex-focused parenting (parental values and communication that are directly concerned with sexual matters) and generic parenting processes (day-to-day parent-teenager relationship and parental supervision) may both be associated with a teenager’s sexual behavior.

Reviews of empirical studies of sex-focused parenting have found consistent associations between parental values and reduced sexual risk-taking, albeit more mixed findings on possible associations with parental communication about sex.^1^–^5^ In part, the mixed findings for communication about sexual risk may reflect methodological limitations.^2^ Limited research indicates that parental communication regarding sexual risk-taking may promote condom use self-efficacy,^21^ enhanced partner communication skills^22^ and abstinence values.^23^ Thus, through communication, parents might influence a teenager’s sexual norms and beliefs, as well as shape skills for negotiating sexual situations. Parental sexual values also may be conveyed less directly, and teenagers’ perceptions of these values may rely more on implicit and nonverbal transmission than on direct parental communication.^24^ Parental values promoting abstinence would be expected to endorse sex within an established relationship.^25^ The likely effect of parental values promoting contraceptive use is less clear-cut: As highlighted by the tensions over abstinence-only versus comprehensive sex education in the United States, sex educators have long had concerns that promoting contraceptive use may have a normalizing effect, creating the expectation that teenagers will engage in early sexual behavior.^26^

The emerging study of adolescent romantic relationships has drawn on ecological and interpersonal theories of development, suggesting the importance of the parent-child relationship in shaping the context, style and meaning of a teenager’s relationships with individuals outside the family.^27^ One of the most influential models, attachment theory, suggests that supportive relationships with parents enable teenagers to develop interpersonal skills and shape attitudes and beliefs that influence their choices and views of relationships.^28^,^29^ Research points to associations between the quality and style of parent-child relationships and corresponding aspects of adolescent or young adult romantic relationships,^28^,^30^,^31^ including several prospective studies.^32^–^36^ The influences of generic parenting may extend to the quality of sex within romantic relationships, and evidence suggests that parental support is positively associated with adolescent skill in interactions with a sexual partner.^19^ However, in general, the sexual component of teenage romantic relationships is poorly understood,^37^ and research emphasis on these relationships has not been matched by research on the high proportion of teenage sexual encounters that take place with a casual (nonromantic) partner.^38^

A second aspect of generic parenting is parental supervision. Both parental monitoring of teenagers’ spare time and limits on television viewing can delay first sex, although little is known about the mechanisms involved.^3^,^39^,^40^ Parental monitoring might restrict opportunities for casual sex in situations that a teenager cannot readily control, although the effects of supervision on initiation of sexual behavior may go beyond opportunity restriction.^41^ Successful monitoring may reflect greater parental knowledge of a teenager’s activities;^42^ if so, such knowledge may be a proxy for parent-teenager closeness and reflect enhanced channels of communication of parental values and skills. Limits on television viewing might influence teenagers’ values, partly by restricting exposure to sexually explicit content, which is predictive of early sexual initiation and teenage pregnancy,^43^,^44^ and partly by signaling parental sexual values.

## RESEARCH GOALS

Our first research aim is to identify parenting processes that are associated with sexual risk avoidance, autonomy and relatedness. The evidence that parenting (sex-focused or generic) is associated with sexual autonomy and relatedness is more limited than the evidence for associations between parenting and sexual risk. Both types of process may be independently associated with teenage sexual health.^45^–^47^ A clearer understanding of the likely impact of parental values and communication about sex when set in the broader context of generic parenting should improve the design of any parenting component to youth sexuality programs, many of which focus on improving parent-child communication about sexual matters.^48^

Our second research aim is to see if associations between parenting processes and teenagers’ sexual values, attitudes and behaviors vary among females and males. One review^5^ found that although mother-child connectedness was important for adolescents of both genders, parental monitoring and communication about sex had a stronger association for females.

Finally, we aim to explore possible mechanisms underlying any associations between parenting and teenage sexual risk, autonomy and relatedness. These outcomes are interrelated, as delayed first sex is less likely than early initiation to be coerced or with a casual partner.^16^,^18^,^49^ To assess whether any associations between parenting and sexual autonomy and relatedness are connected to parenting processes aimed primarily at delaying intercourse, we examine the mediating effect of age or expected age at first sex on these associations. We also investigate whether associations between parenting and sexual risk, autonomy and relatedness are mediated by teenagers’ attitudes in relation to these outcomes. Attitudes could be associated with several outcomes. For instance, a teenager who wishes to prevent sexual risk may avoid sexual situations that are relatively difficult to control.

## METHODS

### Data

The data were collected in a survey conducted in January–May 2007. The survey involved secondary 4 students (similar to U.S. grade 10), with a mean age of 15.5 years, from 13 schools among four local authorities in central Scotland. It provided the baseline data for an evaluation of Healthy Respect, a national demonstration project funded by the Scottish government to improve young people’s sexual health.^50^ The survey took place a year before seven schools based in two local districts in the east of Scotland (the intervention area) started using an enhanced teacher-delivered sex education package.^51^ Two local districts in the west of Scotland, with rates of teenage pregnancy and abortion that were comparable to those in the intervention area, were selected as the comparison area. Schools in this area were matched to those in the intervention area by the proportion of students eligible for free school meals.

Ethical approval for the research was granted by Napier University’s Acute and Continuing Care Nursing and Community Health Schools’ ethics committee. Permission to administer the questionnaire was granted by the education departments of the local authorities concerned. Parents were informed by letter of the research and had the opportunity to withdraw their children from the survey; none did so. Self-completed, anonymous questionnaires were administered to students by trained researchers under examination conditions. Researchers explained the study to individual classes and answered questions. Students could opt out of the survey or omit questions. Absentees were followed up within each school.

The eligible sample consisted of 2,283 students. Of these, 1,990 (87%) completed questionnaires. Some 270 students were absent on the day the survey was administered and failed to complete a questionnaire on their return to school, and 10 refused to participate. Nine questionnaires were excluded because gender or school information was missing, and four were excluded because according to data cleaning, students had not taken the survey seriously.

### Outcome Measures

#### •Sexual risk

Delayed first intercourse was defined as no sexual intercourse by the time of the survey and was derived from two questions: One asked if teenagers had ever had penetrative sex (anal or vaginal), and the other asked the gender of sexual partner. (A clear definition of penetrative sex—anal or vaginal intercourse—was provided to all students before they started answering any questions.)

This allowed students with a same-sex partner to answer the same questions about first sex as students with an opposite-sex partner. We excluded teenagers reporting a same-sex partner from the analytic sample, but we cannot distinguish experience of anal intercourse from experience of vaginal intercourse.

Lifetime condom use was measured with the question “How often did you or your partner use a condom (for all the times you had penetrative sex)?” Response options were “never,”“not very often,”“about half the time,”“most of the time” and “always.”

#### •Sexual relatedness and autonomy

Two measures of the context of first sex were assessed for all teenagers reporting heterosexual intercourse. Sexual relatedness was an eight-point scale derived from two questions: whether the first sexual partner was a “boyfriend or girlfriend” (coded 1 if yes) and, if so, the duration of this relationship before the couple had sex (seven-point scale with responses ranging from “less than a week” to “over a year”). Sexual autonomy was the mean score of three items concerning agreement with nonautonomous reasons for having sex (“forced,”“too difficult to say no,”“thought he/she would leave me if I didn’t”), all recorded using five-point scales, ranging from “strongly agree” to “strongly disagree” (Cronbach’s alpha, 0.66). This measure was validated using a factor analysis of 10 questionnaire items concerning reasons for first intercourse. Items with cross-loadings were removed, and the three items retained loaded on the same factor (factor loadings were all greater than 0.7).

Relatedness expectation was assessed for sexually inexperienced teenagers, who were asked for the situation in which they envisaged first having penetrative sex. Response options were expecting to be in a relationship (“married,”“engaged” or “going steady”), expecting to be “in love,” expecting to be “sexually attracted” and “don’t know.” We compared three groups—those who expected relationship, romance and sexual attraction. Those who said “don’t know” did not differ in social and demographic or academic characteristics from those who gave an expectation and were excluded.

### Independent Measures

#### •Generic parenting processes

Parental supportiveness was measured using standardized mean scores of eight items (Cronbach’s alpha, 0.78). Seven items asked the extent to which teenagers agreed that their parents*“sense when I’m upset about something,”“encourage me to talk about my difficulties,”“are loving,”“trust my judgment,”“don’t understand what I’m going through these days,”“try to control everything I do” and “treat me like a baby”; responses ranged from “strongly agree” to “strongly disagree.” One item asked for the frequency of “serious disagreements or arguments” with parents “about, for instance, drinking, your friends, homework, tidiness or what you wear”; responses ranged from “every day” to “never.” All answers were coded using a five-point scale, and the last four items were reverse-coded.

Parental monitoring was measured using six items: the extent to which teenagers have to ask permission to go out, be home by a certain time and telephone and text parents about a change in plans, and to which parents stay up until the teenager returns home and know of the teenager’s whereabouts (Cronbach’s alpha, 0.71). Standardized mean scores were calculated from responses on a four-point scale (“always,”“usually,”“sometimes” or “never”).

Teenagers were considered to be subject to parental rules about TV or DVD content if they agreed with any of these three statements: “I am not allowed to watch some programs,”“I am not allowed to watch 18 certificate films,”† and “I am not allowed to watch DVDs or TV with a lot of sex.” Responses to these statements were supplemented by information from free text responses to a question inviting teenagers to describe “other rules.” Teenagers were classified as having no rules about TV content if they agreed with the statement “I can watch anything I like” or agreed that their parents placed restrictions on the amount or timing of their TV viewing (“I’m only allowed to watch a certain amount of TV” or “I have to finish my homework or special jobs first”). We combined these two groups after exploratory analysis found no difference in the association with sexual outcomes.

#### •Sex-focused parenting processes

Perceived parental values restricting sexual intercourse were measured by asking all teenagers whether they thought that each of their parents would agree that “people should be in a loving relationship before having penetrative sex” and “would disapprove of me having penetrative sex.” The four items were coded on a five-point scale from “strongly disagree” to “strongly agree,” and standardized mean scores were calculated (Cronbach’s alpha, 0.71). Perceived parental values endorsing contraceptive use were measured in a similar way, using standardized mean scores for responses to two statements: “My mother/father would want me to use condoms if I had penetrative sex” and “My mother/father would want me/my girlfriend to go on the pill if I had penetrative sex” (Cronbach’s alpha, 0.78).

Frequency of parental communication was measured from 11 items about communication with parents (of either gender) about sexual situations and contraception during the past year. Communication about sexual situations covered six topics: “whether it is a good idea to have a serious boyfriend or girlfriend at your age,”“places they don’t want you to go,”“people they don’t want you to go out with,”“how to behave with a boyfriend or girlfriend,”“situations that could lead to having sex with someone you don’t want to” and “when it is ok to have penetrative sex.” Communication about contraception covered five topics: “contraception (ways to avoid pregnancy when having sex, e.g., condoms, the pill),”“how to use condoms properly,”“where to get advice about contraception,”“getting pregnant/getting someone pregnant” and “how to avoid sexually transmitted infections.” Frequency of communication was measured using a four-point scale (“not at all,”“just a little,”“quite a lot,”“very often”), and standardized mean scores were calculated (Cronbach’s alpha, 0.89).

Ease of parental communication consisted of standardized mean scores of two items measuring how comfortable teenagers felt when talking with their mother and father about sex, using a five-point scale from “very uncomfortable” to “very comfortable” (Pearson correlation, 0.38; p<.001).

#### •Hypothesized mediators for parenting processes

Age in years at first sex was used for sexually experienced teenagers, and anticipated age at first sex was used for sexually inexperienced teenagers (five-point scale from “by the age of 16” to “when 20 or older,” excluding teenagers who said “don’t know”). The “don’t know” group was similar in composition to teenagers who gave an age.

Attitudinal mediators were assessed for all teenagers, regardless of sexual experience. Condom use intentions were standardized mean scores for five items asking about intentions to get, carry, discuss using, suggest using and always use condoms. Responses were coded on a five-point scale from “do not strongly intend” to “strongly intend” (Cronbach’s alpha, 0.75). Autonomy intention was the mean score of responses to two statements indicating intention: “to say no to doing something sexual you don’t want to do” and “if things become sexual I intend to tell my partner exactly how far I want to go.” Responses were coded on the same five-point scale (Pearson correlation, 0.26; p<.001). Relatedness value was derived from agreement with two statements: “People should only have penetrative sex within a loving relationship” and “casual sex is acceptable if a condom is used.” A five-point scale from “strongly agree” to “strongly disagree” was used (Pearson correlation, 0.35; p<.001). After the first item was reverse-coded, mean scores were calculated.

### Analysis

Bivariate analyses explored correlations between parenting measures and associations between parenting measures and experience of sexual intercourse.

Multivariate models examined the association of parent-ing measures with sexual outcomes using Stata version 10, taking account of clustering by school. In the first step, covariates in all models consisted of gender, age in months at interview, study area (intervention or comparison), father’s education (whether the teenager’s father left school at age 16, the minimum allowable age), family structure (whether the teenager lived with both biological parents) and academic ability (number of Scottish standard grade subjects studied at credit level).* Models used linear regression, except for first sex (binary logit), condom use (ordered logit) and relatedness expectation (multinomial regression). Associations are reported as statistically significant where p<.05.

We initially performed complete case analyses. In all models, levels of missing information for outcomes and covariates were elevated among teenagers whose father had left school at age 16, were relatively young at the time of interview, were studying for fewer standard grades at credit level, expected to leave school at age 16, reported low parental monitoring and reported heterosexual intercourse. To decrease bias and increase the power of the analyses, we used multiple chained equations (ICE program, version 1.7.0) to impute missing values.^52^ Reduction in bias is expected when the items to be imputed are missing at random, meaning that their values are comparable with those observed for each variable, given the observed values of other variables used in the imputation model. The clustering of students by school was ignored in the imputation for simplicity. We generated 20 imputed data sets, and estimates were combined across these using the *micombine* procedure in Stata.^53^,^54^ Results were similar to those from complete case analyses, and for simplicity, we present only results using the imputed data set here.

In the second step, interactions between gender and parenting measures were added to the models, to test for gender differences in associations.

In the third step, we investigated possible mediators for parenting processes that were associated with delayed first intercourse (among all teenagers); condom use, sexual autonomy and sexual relatedness (among sexually experienced teenagers); and relatedness expectation (among sexually inexperienced teenagers). We followed the three-stage approach recommended by Baron and Kenny.^55^ We regressed each parenting measure and the covariates on each proposed mediator. We then regressed the parenting measure and covariates on the outcome measure, and repeated this calculation including the proposed mediator as an independent variable. Mediation occurs when three conditions are met: The parenting measure is associated with the mediator, the mediator is associated with the sexual outcome, and the association between the parenting measure and the sexual outcome loses significance or shrinks upon the addition of the mediator to the model. We conducted a Sobel test to establish whether a given mediator carried the influence of a parenting measure on the outcome. Where Sobel tests were significant at p<.05 for at least one combination of parenting process and mediator, we added mediators to full models with all parenting measures as covariates. We calculated the indirect effect of parenting via mediators as a proportion of the total effect (i.e., the reduction in the size of a coefficient for a parenting measure after mediators are included).

## RESULTS

### Descriptive and Bivariate

In all, 1,990 students returned usable questionnaires. Of these, 1,854 teenagers (926 males and 928 females) provided information on whether they had experienced penetrative intercourse; this group formed the base sample for our analysis. The sample was mostly white; only 6% were from ethnic minority groups (Table 1, page 33); 31% did not live with both biological parents, and 38% had a father who had left school at age 16.

**TABLE 1:** Selected characteristics of students participating in a sexual health intervention evaluation, by gender, Scotland, 2007

Characteristic	Total	Male	Female
ALL	(N=1,854)	(N=926)	(N=928)
**Social and demographic**			
Mean age (mos.)	195.86 (16.61)	195.59 (16.13)	196.13 (17.07)
Nonwhite*	6	7	5
Do not live with both biological parents	31	32	30
Father left school at 16	38	38	38
No. of standard grade subjects studied			
8	43	41	44
6–7	20	22	18
≤5	37	37	37
**Sexual behavior**			
Ever had sexual intercourse**	32	29	35
Median age at first intercourse (range, 10–16)	14	14	14
SEXUALLY EXPERIENCED	(N=592)	(N=269)	(N=323)
**Condom use**			
Never	12	13	11
Not very often	10	8	12
About half the time	8	8	8
Most of the time	19	14	22
Always	51	57	47
Mean sexual autonomy at first sex (range, 1–5)***	4.07 (0.69)	3.93 (0.65)	4.18 (0.69)
Sexual relatedness at first intercourse***			
No relationship	27	32	23
Relationship of <1 week	3	3	3
Relationship of 1–2 weeks	5	6	4
Relationship of 2 weeks–1 month	11	13	10
Relationship of 1–3 months	20	20	20
Relationship of 3–6 months	18	14	22
Relationship of 6 months–1 year	11	8	13
Relationship of >1 year	5	4	6
SEXUALLY INEXPERIENCED	(N=1,262)	(N=657)	(N=605)
Expected context of first intercourse**			
In relationship†	23	20	26
In love	19	15	22
Sexually attracted	20	26	13
Don't know	39	39	39

* p<.05.

** p<.01.

*** p<.001.

† Married, engaged or going steady.

*Notes:* Unless otherwise noted, data are percentages. Figures in parentheses are standard deviations. Measures used for mean scores are not standardized. Percentage distributions may not add to 100 because of rounding. Chi-square or t tests were used to assess significance of gender differences.

At the time of the survey, 32% of the base sample—35% of females and 29% of males—had had heterosexual intercourse. The median age at first intercourse was 14 in both sexes. Although condom use did not vary by gender, other sexual outcomes did. Females reported greater autonomy than males at first sex (means, 4.2 and 3.9, respectively), and were more likely than males to have experienced first sex within the context of a relationship (77% vs. 68%) or to anticipate doing so (26% vs. 20%).†

Correlations between many of the parenting measures were low but significant. For example, parental values restricting intercourse were positively associated with parental monitoring (r=0.27) and supportive relationship (0.21), and parental values promoting contraceptive use were positively associated with frequency of parental communication (0.25). These correlations suggest that sex-focused parenting and generic parenting measures are interrelated to some extent and underline the importance of including a range of measures in the multivariate models. (Full results are available on request.)

Analyses of sex-focused parenting revealed low overall frequency of parental communication about sex (mean, 1.6—Table 2), although parental values reported by all teenagers were relatively high (3.5 for restricting intercourse and 4.0 for endorsing contraception). Compared with sexually experienced teenagers, those without sexual experience reported less parental communication about sex (1.5 vs. 1.9) and lower parental endorsement of contraceptive use (3.9 vs. 4.1). However, sexually inexperienced teenagers reported a more supportive parental relationship than their sexually experienced peers (3.7 vs. 3.6), as well as more parental monitoring (2.9 vs. 2.7) and parental restrictions on TV content (33% vs. 16%).

**TABLE 2:** Measures of selected sex-focused and generic parenting processes, by teenagers' sexual experience

Parenting process	All	Sexually inexperienced	Sexually experienced
**Sex-focused**			
Frequency of parental communication (range, 1–4)***	1.64 (0.59)	1.54 (0.54)	1.85 (0.64 )
Ease of parental communication (range, 1–5)	2.31 (1.06)	2.29 (1.05)	2.33 (1.08)
Parental values restricting intercourse (range, 1–5)	3.46 (0.68)	3.48 (0.67)	3.42 (0.70)
Parental values endorsing contraceptive use (range, 1–5)***	3.95 (0.68)	3.89 (0.69)	4.07 (0.66)
**Generic**			
Parental supportiveness (range, 1–5)***	3.65 (0.66)	3.69 (0.63)	3.58 (0.71)
Parental monitoring (range, 1–4)***	2.80 (0.60)	2.85 (0.60)	2.70 (0.59)
Parental rules about TV content (%)***	27	33	16

*** p<.001.

*Notes:* Unless otherwise noted, data are unstandardized means (and standard deviations).

Chi-square or t tests were used to assess significance of gender differences.

### Multivariate

Parental communication about sex was not associated with any sexual outcomes except delayed intercourse (Table 3). Frequency of communication was negatively associated with delayed intercourse (beta, –0.5), and greater ease of parental communication about sex was positively associated with delayed intercourse (0.1). In contrast, parental values were associated with a range of outcomes. Values restricting intercourse were positively associated with delayed intercourse, sexual autonomy, sexual relatedness and sexually inexperienced teenagers’ expectation that first sex will occur when they are in love or in a relationship, rather than just sexually attracted to someone (0.1–0.5). However, the more strongly students agreed that their parents endorsed contraceptive use, the less likely they were to delay intercourse and the more likely they were to expect first sex when sexually attracted, rather than when in love or in a relationship (–0.2 to –0.5).

**TABLE 3:** Coefficients from multivariate analyses assessing associations between parenting processes and sexual outcomes, by teenagers' sexual experience

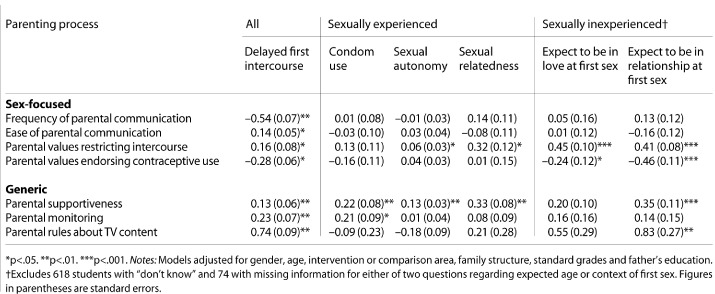

In the generic parenting category, parental supportiveness was positively associated with all outcomes except expecting to be in love rather than sexually attracted at first sex (betas, 0.1–0.4). Parental monitoring was positively associated with delayed intercourse (0.2) and condom use (0.2), while rules about TV content were associated with delayed sex (0.7) and expecting first sex to occur in a relationship, rather than simply because of sexual attraction (0.8).

The associations between parenting measures and teenage sexual behaviors, experiences and expectations that were obtained by adding a term for the interaction of gender and parenting to the Table 3 models revealed few significant gender differences (not shown). In the model of condom use, parental monitoring was significant for females (beta, 0.4), and frequency of parental communication was significant for males (–0.4). In the sexual autonomy model, the interaction coefficient for females with parental monitoring bordered on significance (0.1; p<.10).

### Mediators

Some associations between parenting and sexual behavior were mediated by older age at first sex or expected age at first sex (Table 4, page 36). In the model of condom use, age at first sex reduced the coefficients for supportive relationship (from 0.22 to 0.20) and parental monitoring (0.21 to 0.19), in both cases to nonsignificance. In the model of expecting first sex to occur in a relationship expected age at first sex had a similar effect on TV rules (0.8 to –0.4).

**TABLE 4:** Coefficients from multivariate analyses assessing associations between parenting processes and sexual outcomes, with and without adjustment for potential mediators, and estimated indirect associations via mediators

Outcome and parenting process	No adjustment for mediators	Adjusted for age at first sex/expected age first sex	Adjusted for strongest attitudinal mediator	Adjusted for all mediators	Indirect association via mediators (%)
**Delayed first intercourse**†					
Frequency of parental communication	−0.54 (0.07)**	.na	–0.55 (0.07)***	–0.55 (0.07)***	0
Ease of parental communication	0.14 (0.05)*	.na	0.13 (0.05)**	0.13 (0.05)**	7
Parental values restricting intercourse	0.16 (0.08)*	.na	0.07 (0.09)	0.07 (0.09)	56
Parental values endorsing contraceptive use	–0.28 (0.06)*	.na	–0.25 (0.06)***	–0.25 (0.06)***	11
Parental supportiveness	0.13 (0.06)**	.na	0.10 (0.06)	0.10 (0.06)	23
Parental monitoring	0.23 (0.07)**	.na	0.21 (0.07)**	0.21 (0.07)**	9
Parental rules about TV content	0.74 (0.09)**	.na	0.69 (0.09)***	0.69 (0.09)***	7
**Condom use**‡					
Parental supportiveness	0.22 (0.08)**	0.20 (0.08)	0.16 (0.08)	0.13 (0.09)	41
Parental monitoring	0.21 (0.09)*	0.19 (0.09)	0.09 (0.09)	0.07 (0.09)	67
**Sexual autonomy**§					
Parental values restricting intercourse	0.06 (0.03)*	.na	.na	0.06 (0.03)*	0
Parental supportiveness	0.13 (0.03)**	.na	.na	0.13 (0.03)**	0
**Sexual relatedness**††					
Parental values restricting intercourse	0.32 (0.12)*	.na	0.19 (0.12)	0.19 (0.12)	41
Parental supportiveness	0.33 (0.08)**	.na	0.30 (0.09)**	0.28 (0.09)*	12
**Expect first sex when in love**‡‡					
Parental values restricting intercourse	0.45 (0.10)***	0.26 (0.11)*	0.26 (0.12)*	0.10 (0.12)	78
Parental values endorsing contraceptive use	–0.24 (0.12)*	–0.15 (0.14)	–0.14 (0.14)	–0.12 (0.16)	50
**Expect first sex when in relationship**‡‡					
Parental values restricting intercourse	0.41 (0.08)***	0.19 (0.09)*	0.22 (0.10)*	0.05 (0.11)	88
Parental values endorsing contraceptive use	–0.46 (0.11)***	–0.34 (0.15)*	–0.35 (0.12)**	–0.31 (0.15)*	33
Parental rules about TV content	0.83 (0.27)**	0.43 (0.28)	0.68 (0.27)*	0.31 (0.30)	63
Parental supportiveness	0.35 (0.11)***	0.30 (0.14)*	0.33 (0.12)**	0.25 (0.15)	29

* p<.05.

** p<.01.

*** p<.001.

† Mediated by relatedness value.

‡ Mediated by age at first sex and (more strongly) condom intentions.

§ No mediators.

†† Mediated by age at first sex, autonomy intentions and (most strongly) relatedness value.

‡‡ Mediated by expected age at first sex, autonomy intentions and (most strongly) relatedness value.

*Notes:* All models adjusted for the same covariates listed in Table 3. na=not applicable. Figures in parentheses are standard errors.

Attitudinal variables also acted as mediators. Condom use intentions were the strongest mediator of parenting in the condom use model, and their inclusion reduced the coefficients for supportive relationship (0.22 to 0.16) and parental monitoring (0.2 to 0.1), to nonsignificance in both cases. Teenagers’ relatedness value reduced the coefficients for parental values restricting intercourse in the models of delayed first sex (0.2 to 0.1) and sexual relatedness (0.3 to 0.2), in both cases to nonsignificance. It partially mediated other associations; for example, when it was added to the model of delayed intercourse, the coefficient for TV rules was reduced but still statistically significant. Relatedness value also partially mediated some of the negative association with parental values endorsing contraceptive use, since teenagers who reported high parental contraceptive values placed lower value on having sex in the context of a meaningful relationship. For example, the coefficient for parental contraceptive values in the model of expecting first sex in a relationship was reduced from –0.5 to –0.4 but remained significant. Autonomy intentions did not mediate any associations between parenting and sexual autonomy or any other outcomes.

Overall, except in the models of sexual autonomy (which had no mediators) and delayed first sex (which had only one), adjustment for mediators left few significant direct associations with parenting. Indirect associations between parenting and sexual outcomes via mediators varied considerably. For parental monitoring and TV rules, adjustment for all mediators reduced the overall coefficients by 7–67%. For supportive parenting, reductions were in the range of 12–41%, and for parental values restricting intercourse, 41–88%.

## DISCUSSION

The study suggests that parenting may be associated with multiple benefits to teenagers’ sexual relationships—delayed intercourse and greater condom use, as well as greater sexual autonomy and an increased likelihood that sex will occur within a relationship, which other research has indicated are associated with higher quality sexual experiences.^16^–^18^ Thus, the benefits of parenting may extend beyond helping teenagers avoid sexual risk, enhancing their capacity to have positive early sexual relationships.

A supportive parent-teenager relationship and parental values restricting intercourse had the most pervasive positive associations with sexual outcomes. Their association with sexual risk-taking is broadly in line with those observed in previous research;^1^–^5^ however, we found no association between parental intercourse values and condom use, and earlier findings for parental supportiveness have been inconsistent.^1^–^4^ We also found positive associations between these two dimensions of parenting and sexual relatedness and autonomy. The relatively small association between parental values restricting intercourse and sexual autonomy could be an indirect result of associations with relatedness, since having first sex within a relationship instead of casually makes it easier for teenagers to exercise autonomy.^16^ Overall, the associations between parenting and sexual relatedness and autonomy did not entirely depend on teenagers’ age at first sex or expected age at first sex, as would be predicted if they simply resulted from associations between parenting and sexual risk-taking. Associations between parental intercourse values and relatedness echo findings from a U.S. study, based on the National Longitudinal Study of Adolescent Health, in which parental values affected teenagers’ selection of a romantic rather than a casual partner, and were largely mediated by the value teenagers placed on having sex in a loving relationship.^38^ However, the basis for associations between a supportive relationship and sexual autonomy and relatedness in our study did not appear to be confined to a narrow range of influences on teenagers’ values and intentions. Our findings are in line with theoretical approaches suggesting that parents may shape teenagers’ social skills and attitudes toward sexual relationships, and with research indicating that parental support is positively associated with teenagers’ sense of volitional functioning and adjustment.^12^,^56^

Parental monitoring and rules restricting TV content had less pervasive associations with sexual outcomes. Significant findings for parental monitoring were restricted to associations with risk avoidance—delayed sex and condom use—and generally echo previous results.^1^–^4^ For some outcomes, monitoring appeared to have a stronger association in females than in males, as earlier research in Scotland has shown.^57^ Parental rules restricting TV content were associated with delayed first sex (in line with other research^39^,^45^) and anticipating sex in a relationship. In part, these associations may be explained in terms of the value teenagers placed on relationship sex. This suggests that media influences (from TV and other sources, including the Internet) on sexual norms and expectancies, which some researchers have begun to investigate in relation to teenagers’ initiation of sexual behavior,^58^–^61^ should be explored in more detail. Alternatively, TV rules might be a proxy for general parental attitudes toward sex.

Greater ease of communication was associated with delayed first sex, as reported elsewhere.^24^,^45^ However, some negative associations with sexual outcomes were found for both parental values endorsing contraceptive use and frequency of parental communication. For contraceptive values, this might simply reflect greater certainty over parental approval on the part of teenagers who are either anticipating or already engaged in sexual activity. Similarly, the negative association between more frequent communication and delayed first sex could reflect reverse causation in the cross-sectional data, if anticipated or actual first sex leads to increased communication.^62^–^64^ Or, more frequent discussion could signal liberal parental attitudes toward early sex. We found that parental approval of contraceptive use was negatively associated with the value teenagers placed on having sex while in a meaningful relationship. This could suggest that teenagers link parental advocacy of contraceptive use with more permissive attitudes toward casual sex, as other research suggests,^65^ or that a teenager’s permissive attitudes prompt parents to express approval of contraceptive use. In prospective studies, perceived parental approval of contraceptive use and more frequent communication have predicted sexual initiation.^45^,^66^

### Limitations and Strengths

Our analysis has a number of limitations, notably the use of teenagers’ self-reported data and the cross-sectional design. Questions about the validity of self-reported data on sexual behavior, and possible discrepancies between teenagers’ and parents’ reported perceptions of parenting, are common issues in studies of this type. However, studies collecting data from both parents and teenagers have suggested that teenagers’ perceptions are better predictors of outcomes than parents’ responses.^67^ The associations we found may reflect not causal links, but the operation of other, unmeasured influences. These could include other parenting processes (for example, parents’ overall expectations for their child may influence perceived sexual values^68^), as well as other aspects of the teenager’s social environment, such as peer group and school.

The cross-sectional nature of the study, as already noted, precludes our assessing the direction of causation. Teenagers engaged in risky sexual activities may develop a poor relationship with their parents and make it more difficult for their parents to regulate their behavior. Negative effects on a teenager’s relationship with parents following first sex have been observed in longitudinal studies.^69^,^70^

Further limitations include the interchangeable use of “parent” and “guardian” in the parenting measures, and the fact that few measures were collected separately for each parent, which further restricted investigation of differences according to whether there was a biological relationship, and according to different parent-child dyads. The largely white sample limits generalizability to ethnic subgroups in which differences in parenting processes in relation to teenage sexual behavior have been noted.^46^,^71^ Our data mainly relate to first intercourse, and parenting influences may wane once teenagers are sexually experienced. Finally, most young people in this study had not yet had sexual intercourse. As teenagers grow older and more independent, the parent-child relationship and parenting processes change.^19^,^72^ Plus, teenagers who start sex later may be subject to different predictors of sexual behavior than those who start early.^3^,^73^

The study also has a number of strengths. The wide range of parenting measures allowed us to capture more of the complex nature of parenting processes than have studies relying on two or three measures. We were able to explore whether teenagers may be influenced by parental communication after considering more generic day--to-day parenting processes and any indirect transmission of parental values. More longitudinal work is needed to investigate possible mechanisms for the associations we found, particularly the role that the parent-child relationship plays in shaping skills required in developing intimate sexual relationships, and to clarify the role of parenting processes in a wider social context (for example, peer and school influences).

### Conclusion

While our study represents only a first step toward under-standing the wider aspects of the relationship between parenting and teenage sexual behavior and cannot confirm causality, it supports the view that both sex-focused and generic parenting may indirectly promote more positive, as well as safer, early sexual experiences. It suggests that parenting may be important in helping teenagers, even those who have sex at an early age, to develop values and skills for managing relationships. It also suggests that educators and health professionals should not frame the parental role solely in terms of advocating delayed sex.

Like many similar studies of sex-focused parenting, ours might lack potentially important details on the content and quality of parental communication about sex.^2^,^74^ The predominant approach of parenting interventions designed to reduce children’s sexual risk behaviors is to promote sex-specific parental communication.^21^ Our findings suggest that this will be neither simple nor sufficient for promoting more positive sexual development. Programs should acknowledge that parents may transmit values restricting intercourse over an extended period, and through indirect means, not only through “sex talk” with their teenagers. However, possible tension between parental messages about delaying sex and promotion of contraceptive use for teenagers who are having intercourse must be resolved.

Generic parenting is likely to have a much longer period of influence than sex-focused parenting. Although parenting programs incorporating generic approaches have promoted parental monitoring,^75^,^76^ another form of supervision, parental restriction of TV content, deserves wider attention. Interventions also should focus on the importance of a supportive parent-teenager relationship, which factors into teenagers’ lives several years before they are likely to start sexual activity.

## Footnotes

* The World Health Organization defines sexual health as “a state of physical, mental and social well-being in relation to sexuality. It requires a positive and respectful approach to sexuality and sexual relationships, as well as the possibility of having pleasurable and safe sexual experiences, free of coercion, discrimination and violence.” (Source: World Health Organization, Sexual health, no date, <http://www.who.int/-topics/-sexual_health/en/>, accessed Oct. 25, 2010.)

* Throughout the text, “parent” also denotes “guardian.”

† These are films classified by the British Board of Film Classification as not to be viewed, rented or purchased by those younger than 18.

* Standard grades are Scotland’s educational qualifications, examined at ages 15–16 (broadly equivalent to the General Certificate of Secondary Education taken in other parts of the United Kingdom). Within the standard grade system, examinations are set at three levels of difficulty: foundation, general and credit (highest academic) level. Not all students will study a subject to credit level; some may sit only for foundation- or general-level examinations.

† The autonomy measure also was associated with partner pressure to have sex (not shown). Although only a minority reported partner pressure, females were more likely than males to do so (24% vs. 11%, p<.001). Other aspects of volition, such as perceived control over sexual desires, may account for females’ higher sexual autonomy scores.
